# Overweight and obesity among female adolescents in Nigeria; an emerging, but under-reported epidemic

**DOI:** 10.1186/s12905-024-03146-4

**Published:** 2024-05-21

**Authors:** Adeleye Abiodun Adeomi, Nafisat Olayinka Olatunde Lawal

**Affiliations:** Department of Community Health, Obafemi Awolowo University Teaching Hospitals Complex, Ile-Ife, Osun State Nigeria

**Keywords:** Obesity, Overweight, Female adolescents, Determinants, Nigeria

## Abstract

**Background:**

Overweight and obesity are increasing at epidemic levels in all ages globally, but there is little nationally representative data on female adolescents in Nigeria. The focus on female adolescents is important because of the negative implications of overweight and obesity on their health and survival, and that of their unborn children.

**Aim:**

To estimate the prevalence and identify the determinants of overweight and obesity among female adolescents in Nigeria.

**Methods:**

Cross-sectional study using data from the Nigeria demographic and health survey, 2018. A total of 2,721 female adolescents aged 15–19 years were selected using cluster sampling technique. Overweight and obesity were determined using BMI-for-age reference values of World Health Organization and different explanatory variables at the individual, household and community levels were included. Binary logistic regression analysis was used to identify the determinants of overweight/obesity using five models.

**Results:**

The mean age of the respondents was 16.8 ± 1.4 years. The prevalence rate of overweight/obesity was 10.2%, but with a large variation in the geographical and socio-economic distribution. At the crude/unadjusted rate, nearly all the explanatory variables showed a statistically significant association with overweight and obesity, but at the full model which controlled for all the explanatory variables, only the household wealth index retained its statistically significant association, such that female adolescents who were from richer and richest households had about 3 times higher odds of being obese compared to those from the poorest households. (OR: 2.7; *p* = 0.018; CI: 1.18–6.18), (OR: 2.8; *p* = 0.027; CI: 1.13–7.06) respectively.

**Conclusion:**

The prevalence of overweight/obesity among female adolescents in Nigeria was 10.2%. The household wealth index remained the only factor with a statistically significant association with overweight and obesity after controlling for confounders. Efforts at addressing overweight and obesity among female adolescents in Nigeria should target those from the richer/richest households.

## Background

The prevalence of overweight/obesity is rapidly increasing in the low-and-middle-income countries (LMIC), particularly in Africa [1, 2]. For more than four decades, the prevalence of overweight/obesity among adolescents has been increasing rapidly globally, and more importantly in Africa [3, 4]. Research shows that between 1975 and 2016, the prevalence of obesity among children and adolescents (5–19 years) increased by 450% from 4 to 18% [5]. A study conducted among adolescents in seven African countries reported the prevalence of obesity to range from 0.6 to 9.3% [6]. Obesity is linked to more deaths worldwide than under-nutrition and has been projected to become the leading nutritional cause of death among persons in LMIC by 2030 [2]. According to the World Health organization (WHO), Africa is facing an emerging public health problem of obesity, with the prevalence among children and adolescents ranging from 5 to 16.5% [7]. 

About 20% of the world’s populations are adolescents (10–19 years) and more than 85% of them live in LMIC [8, 9]. In Nigeria, about a quarter of the population i.e. an estimated 40 million people are adolescents [10, 11]. Obese adolescents are more likely to become obese adults hence, have higher odds of developing type-2 diabetes and cardiovascular disease. Adolescent overweight/obesity can result in bullying, discrimination and stigmatization with consequent poor school performance and quality of life [12]. Economically, the impact of overweight/obesity is projected to increase from a global gross domestic product (GDP) of 2.19% in 2019 to 3.29% with a global cost more than US$18 trillion by 2060 [13, 14] if current trend continues.

A major challenge with the existing data on adolescent overweight and obesity in Nigeria is the lack of National representativeness. This is particularly important because Nigeria is a very diverse country with socio-economic inequalities across different regions and groups [15]. ^,^ [16] The efforts made at estimating a national prevalence from the existing scattered data has been challenging as different methodologies and reference values were used [17]. Furthermore, only little is known about the determinants. Much of the efforts at understanding the determinants have targeted few individual factors alone [18], and little about household and community factors. Targeting only individual factors may lead to over-estimation of statistical significance, and thus provide erroneous evidence. Understanding the determinants at individual, household and community levels will help to generate more accurate estimates of association and to better understand the roles of the different contextual units, and these will help in designing interventions that are effective in halting/reducing the rising prevalence of adolescent overweight and obesity in Nigeria [18]. 

The focus on female adolescents is important, firstly because existing studies in Nigeria and sub-Saharan Africa show that female adolescents have a higher burden of overweight and obesity compared to their male counterparts [19–21]. Overweight/obese female adolescents are at higher risk of developing cardiovascular disease, type-2 diabetes, digestive, neurological disorders etc [12]. . Additionally, an obese female adolescent is more likely to be an obese adult [22], which has been found to have negative implications on child birth and survival, and maternal mortality [23, 24]. This study therefore aimed to estimate the prevalence, and identify the determinants of overweight and obesity among female adolescents in Nigeria.

## Methodology

### Study setting and design

This study used data from the NDHS, 2018 [25], which was a community-based Nationally representative survey, using representative sample from all parts of Nigeria. An added advantage of the NDHS data is that it contains possibly the most comprehensive Nigerian data on individual, household and community factors which makes it possible to identify determinants of female adolescent obesity at these levels. The federal republic of Nigeria (simply called Nigeria) is made of six geo-political zones, with three in the north (Northwest, Northeast and North central) and the other three in the south (Southwest, Southeast and Southsouth). There are a total of 36 states and the Federal Capital Territory, which is the capital of Nigeria. Each of the states is divided into local government areas (LGAs), with a total of 774 LGAs in Nigeria. However, enumeration areas (EAs) were created during the National census of 2006, which are clusters/communities within each of the LGAs. A total of 1,400 clusters/communities were randomly selected across the whole country for the 2018 NDHS and used as the primary sampling units (PSUs) [25].

### Sample size and sampling

The sample size for this study was 2,721, which includes non-pregnant female adolescents aged 15 to 19 years. Adolescents between 10 and 14 years were not included because the NDHS has the anthropometric data for 15–19-year-olds alone. Respondents were selected using the two-stage stratified cluster sampling technique. At the first stage, a stratified sample of EAs is selected using proportional allocation, and 20–30 households are selected from each of the selected EAs at the second stage using systematic sampling technique. Sampling weights were applied in the data analysis as appropriate to account for the non-proportional allocation of the sample to the different sub-regions and communities. The research instruments and data collection methods have been further explained in greater details in the NDHS report [25]. 

### Outcome variable

The outcome/dependent variable for this study is overweight and obesity, which were assessed using the BMI-for-age standard deviation (SD). For the present study, adolescents that were either overweight or obese (i.e. those who had > 1 SD) were coded as 1, while others were coded as 0.

### Explanatory variables

The explanatory/independent variables are as explained below.

### Individual characteristics

#### Age

The age was categorized into; middle adolescents (15–16 years) and late adolescents (17–19 years).

#### Educational status

This is the highest level of education completed by the respondents, and it was categorized into no education, primary and secondary or higher and coded as 0, 1 and 2 respectively.

#### Religion

This was categorized and coded into Christian (1), Islam (2) and Others (3).

#### Marital status

Respondent’s marital status was categorized and coded into; not married (0) and married (1).

#### Occupational status

This refers to the occupational status in the 12 months preceding the survey and it was categorized and coded into those who worked (1) and those who did not (0).

#### Minimum dietary diversity

Dietary diversity was measured using the 24-hour dietary recall. Those who ate or drank food types from a minimum of 5 out of the 10 groups were categorized as having minimum dietary diversity and coded as 1, while those with less than 5 were categorized as Others and coded as 0.

### Household characteristics

#### Number of Household members

The usual household members (without the visitors) were categorized into and coded as; 1 to 5 household members (1), 6–10 household members (2), and more than 10 members (3).

#### Household wealth index

This was derived by using principal component analysis to generate a wealth index score from selected household possessions and housing characteristics [25]. Using quintiles, the wealth index score was categorized and coded as into; Poorest (1), Poorer (2), Middle (3), Richer (4), and Richest (5).

#### Community characteristics

##### Region

This was categorized and coded as North central (1), North east (2), North West (3), South east (4), South South (5), and South west (6).

##### Place of residence

Respondent’s place of residence was categorized into Urban and Rural and coded as 1 and 2 respectively.

##### Community education

The median value for the proportion of female adolescents who had a minimum of secondary education in each primary sampling unit was used to divide them into those with Low level of maternal education and High level of maternal education. Low level of maternal education was coded as 1, while and High level of maternal education was coded as 2.

##### Community wealth level

The median value of the proportion of households in the highest wealth index (i.e. richer and richest households) was used to divide them into those with Low community wealth level and those with High community wealth level. These were coded as 1 and 2 respectively.

### Data analysis

Data were analyzed using STATA version 17. Descriptive analysis was done for all the variables, and tests of association was done at the bivariate level using the chi-squared test. Binary logistic regression analysis was used at the multi-variate level to control for confounders and identify the factors associated with adolescent overweight/obesity. A total of 5 models were fitted, with adolescent overweight/obesity being the dependent variable. The first model (Model 0) was the empty model consisting of the unadjusted rates, Model 1 adjusted for individual characteristics of the adolescents alone, Model 2 adjusted for the household characteristics alone, Model 3 adjusted for the community characteristics alone and Model 4 was the full model that adjusted for all the explanatory variables. Adjusted odd ratios were used at 95% confidence interval, with statistical significance set at *p* < 0.05.

## Results

**Fig. 1:**
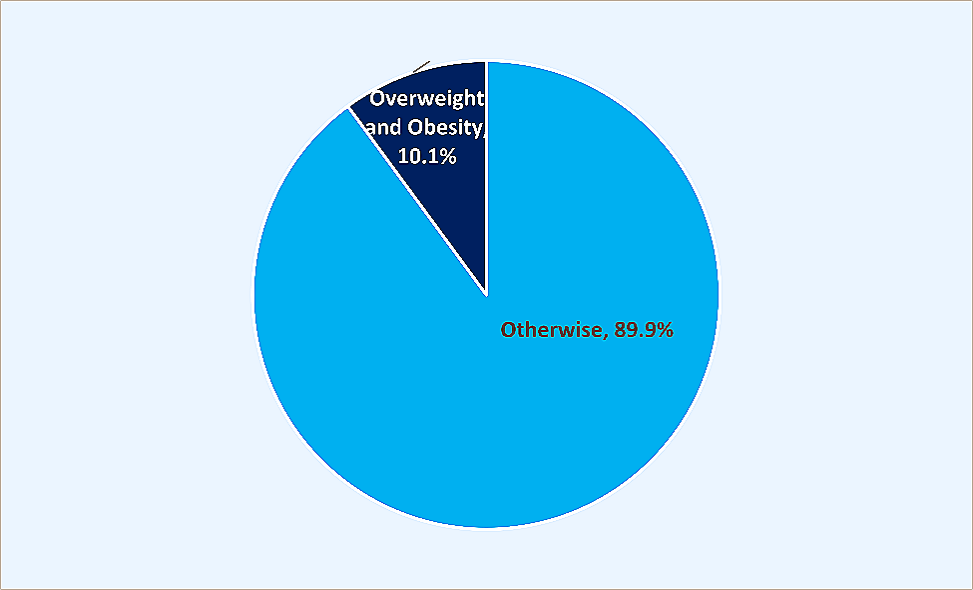
Overall prevalence of adolescent overweight and obesity in Nigeria

Figure 1 shows the prevalence of overweight/obesity among female adolescents and 10.2% of the female adolescents were overweight/obese (Fig. 1). The description of the explanatory variables is shown in Table 1, with 1182 (43.4%) middle adolescents (15–16 years) and 1539 (56.6%) late adolescents (17–19 years), (mean age = 16.8 ± 1.4 years).

**Table 1:** Descriptive statistics of the explanatory variables (*N* = 2,721)

Variables	Frequency	Percent
**Individual Characteristics**		
**Age (in years)**		
Middle adolescents (15–16 years)	1182	43.4
Late adolescents (17–19 years)	1539	56.6
Mean	16.8 ± 1.4 years	
**Educational status**		
No education	683	25.1
Primary	297	10.9
Secondary or higher	1741	64
**Religion**		
Christianity	1127	41.4
Islam	1578	58
^a^Others	16	0.6
**Marital Status**		
Never/formerly in union	2095	77
Currently in union	626	23
**Occupational status (last 12 months)**		
Didn’t work	1672	61.5
Worked	1049	38.5
**Minimum dietary diversity**		
Others	1246	45.8
Minimum dietary diversity	1475	54.2
**Household Characteristics**		
**Number of usual household members (n = 2693)**		
1–5	1066	39.6
6–10	1207	44.8
> 10	420	15.6
**Household wealth index**		
Poorest	466	17.1
Poorer	552	20.3
Middle	586	21.5
Richer	570	21
Richest	547	20.1
**Community Characteristics**		
**Place of residence**		
Urban	1218	44.8
Rural	1503	55.2
**Region**		
North central	391	14.4
North east	527	19.4
North west	834	30.6
South east	298	10.9
South south	307	11.3
South west	364	13.4
**Community educational level**		
Low	1408	51.7
High	1313	48.3
**Community wealth level**		
Low	1370	50.4
High	1351	49.6

^*a*^ *The others here represent traditional worshippers and atheists*

At the bivariate level, prevalence of overweight and obesity was significantly higher among respondents who had secondary/higher education (13.4%, *p* < 0.001), Christians (13.8%, *p* < 0.001) those who were never/formally in a union (11.3%. *p* = 0.002), richest households (17%, *p* < 0.001), urban residence (14%, *p* < 0.001), South-south region (16.9%, *p* < 0.001), communities with high level of education (13.9%, *p* < 0.001) and communities with high wealth level (14.8%), compared to others. (Table 2).

**Table 2:** Distribution of the explanatory variables across overweight/obesity categories (*N* = 2721)

	Otherwise		Overweight /Obese		*N*	Statistics
	%	95% CI	%	95% CI		
**Individual Characteristics**						
**Age (in years)**						
15–16 years	89.4	87.1, 91.4	10.6	8.6, 12.9	1,182	x^2^= 0.5253
17–19 years	90.2	87.8, 92.3	9.8	7.7, 12.2	1,539	*p* = 0.616
**Educational status**						
No education	95.8	93.5, 97.3	4.2	2.7, 6.5	683	x^2^= 58.8669
Primary education	95.5	92.3, 97.4	4.5	2.6, 7.7	297	***p*** < 0.001*
Secondary or higher	86.6	84.3, 88.6	13.4	11.4, 15.7	1,741	
**Religion**						
Christianity	86.2	83.7, 88.4	13.8	11.6, 16.3	1,128	x^2^= 29.2082
Islam	92.5	90.2, 94.3	7.5	5.7, 9.8	1,578	***p*** < 0.001*
Others	89.9	70.0, 97.1	10.1	2.9, 30.0	16	
**Marital Status**						
Never or formerly in union	88.7	86.8, 90.3	11.3	9.7, 13.2	2,095	x^2^= 14.6861
currently in a union	93.9	91.2, 95.8	6.1	4.2, 8.8	626	***p*** = 0.002*
**Occupational status (last 12 months)**						
Otherwise	90.1	88.2, 91.6	9.9	8.4, 11.8	1,673	x^2^= 0.1625
Worked	89.6	86.2, 92.2	10.4	7.8, 13.8	1,049	*p* = 0.792
**Minimum dietary diversity**						
No	89.9	86.9, 92.2	10.1	7.8, 13.1	1,246	x^2^= 0.0001
Yes	89.9	87.8, 91.6	10.1	8.4, 12.2	1,475	*p* = 0.996
**Household Characteristics**						
**Number of usual household members (** ***n*** ** = 2693)**						
1–5	90.8	88.4, 92.8	9.2	7.2, 11.6	1,066	x^2^= 4.8594
6–10	88.6	85.9, 90.8	11.4	9.2, 14.1	1,207	*p* = 0.202
> 10	91.6	88.0, 94.2	8.4	5.8, 12.0	420	
**Household wealth index**						
Poorest	97.3	95.6, 98.3	2.7	1.7, 4.4	466	x^2^= 87.6655
Poorer	92.9	89.8, 95.1	7.1	4.9, 10.2	552	***p*** < 0.001*
Middle	92.7	89.6, 95.0	7.3	5.0, 10.4	586	
Richer	84.5	78.8, 89.0	15.5	11.0, 21.2	570	
Richest	83	78.2, 86.9	17	13.1, 21.8	547	
**Community Characteristics**						
**Place of residence**						
Urban	86	83.2, 88.5	14.0	11.5, 16.8	1,219	x^2^= 36.7896
Rural	93	91.3, 94.4	7.0	5.6, 8.7	1,503	***p*** < 0.001*
**Region**						
North Central	90.6	87.4, 93.1	9.4	6.9, 12.6	391	x^2^= 62.9847
North East	94	91.3, 95.9	6.0	4.1, 8.7	527	***p*** < 0.001*
North West	93.8	91.1, 95.7	6.2	4.3, 8.9	834	
South East	84.4	79.8, 88.1	15.6	11.9, 20.2	298	
South South	83.1	76.9, 87.9	16.9	12.1, 23.1	308	
South West	84.4	77.6, 89.4	15.6	10.6, 22.4	364	
**Community educational level**						
Low	93.4	91.0, 95.2	6.6	4.8, 9.0	1,408	x^2^= 40.9649
High	86.1	83.6, 88.2	13.9	11.8, 16.4	1,313	***p*** < 0.001*
**Community wealth level**						
Low	94.5	92.8, 95.8	5.5	4.2, 7.2	1,370	x^2^= 65.9885
High	85.2	82.5, 87.6	14.8	12.4, 17.5	1,351	***p*** < 0.001*

* Statistically significant; CI – confidence interval; x^2^– chi-squared test; p – *p*-value

At the multi-variate level, there were statistically significant associations (*p* < 0.05) between overweight/obesity and most of the explanatory variables in the crude/unadjusted model (Model 0), except for age, occupation, dietary diversity and number of household members. Model 1 adjusted for individual characteristics alone, and at this level overweight/obesity had statistically significant associations with educational status alone, such that female adolescents with secondary/higher educational status had 3 times higher odds of being obese (OR: 3.0; *p* < 0.001; CI: 1.59–5.82) compared to those with less than secondary education. Household wealth index was the only predicator of overweight/obesity in Model 2 which adjusted for household characteristics alone. It was such that respondents from poorer and middle households had about 3 times higher odds for obesity (OR: 2.6; *p* = 0.005; CI: 1.34–4.94), (OR: 2.8; *p* = 0.002; CI: 1.47–5.22) respectively, while those from richer and richest households had 6 and 7 times higher odds for overweight/obesity (OR: 6.4; *p* < 0.001; CI: 3.41–11.84), (OR: 7.2; *p* < 0.001; CI: 3.93–13.07) respectively, compared to those from poorest households. In Model 3 which adjusted for community characteristics alone, rural dwellers had 70% less odds of having obesity compared to urban dwellers (OR: 0.7; *p* = 0.046; CI: 0.52–0.99) while those from wealthier communities had 2 times higher odds for obesity compared to those from less wealthy communities (OR: 1.8; *p* < 0.001; CI: 1.30–2.59). Model 4 is the full model that adjusted for all explanatory variables, the only variable that retained its statistically significant association with overweight/obesity was the household wealth index such that female adolescents who were from richer and richest households had about 3 times higher odds of being overweight/obese compared to those from the poorest households. (OR: 2.7; *p* = 0.018; CI: 1.18–6.18), (OR: 2.8; *p* = 0.027; CI: 1.13–7.06) respectively. (Table 3)

**Table 3:** Determinants of overweight/obesity among female adolescents in Nigeria using logistic regression analysis (*N* = 2721)

	Model 0			Model 1			Model 2			Model 3			Model 4		
	OR	*p*	95% CI	OR	*p*	95% CI	OR	*p*	95% CI	OR	*p*	95% CI	OR	*p*	95% CI
**Individual Characteristics**															
**Age (in years)**															
15–16 years (R)															
17–19 years	0.9	0.616	0.64, 1.30	0.9	0.644	0.64, 1.32							0.9	0.505	0.61, 1.28
**Educational status**															
No education (R)															
Primary education	1.1	0.846	0.54, 2.10	1.0	0.890	0.45, 1.99							0.6	0.291	0.26, 1.49
≥ Secondary	3.5	< 0.001*	2.13, 5.83	3.0	0.001*	1.59, 5.82							1.8	0.122	0.85, 3.98
**Religion**															
Christian (R)															
Islam	0.5	< 0.001*	0.35, 0.73	0.7	0.149	0.47, 1.12							1.0	0.937	0.59, 1.63
Others	0.7	0.610	0.18, 2.72	0.9	0.833	0.24, 3.15							0.8	0.806	0.20, 3.52
**Marital Status**															
Not married (R)															
Married	0.5	0.002*	0.33, 0.78	1.0	0.951	0.60, 1.74							1.2	0.632	0.64, 2.07
**Occupational status**															
Otherwise (R)															
Worked	1.1	0.792	0.72, 1.55	1.1	0.514	0.77, 1.67							1.2	0.405	0.80, 1.71
**Minimum dietary diversity**															
No (R)															
Yes	1.0	0.996	0.69, 1.46	0.9	0.642	0.62, 1.35							0.9	0.516	0.57, 1.32
**Household Characteristics**															
**Number of usual household members (** ***n*** ** = 2693)**															
1–5 (R)															
6–10	1.3	0.171	0.90, 1.82				1.1	0.538	0.79, 1.56				1.1	0.571	0.77, 1.62
> 10	0.9	0.683	0.57, 1.44				0.9	0.548	0.53, 1.40				1.1	0.811	0.63, 1.79
**Household wealth index**															
Poorest (R)															
Poorer	2.7	0.002*	1.45, 5.20				2.6	0.005*	1.34, 4.94				2.0	0.053	0.99, 4.21
Middle	2.8	0.001*	1.50, 5.29				2.8	0.002*	1.47, 5.22				1.6	0.252	0.72, 3.48
Richer	6.6	< 0.001*	3.52, 12.3				6.4	< 0.001*	3.41, 11.84				2.7	0.018*	1.18, 6.18
Richest	7.4	< 0.001*	4.07, 13.3				7.2	< 0.001*	3.93, 13.07				2.8	0.027*	1.13, 7.06
**Community Characteristics**															
**Place of residence**															
Urban (R)															
Rural	0.5	< 0.001*	0.34, 0.64							0.7	0.046*	0.52, 0.99	0.8	0.233	0.56, 1.15
**Region**															
North Central (R)															
North East	0.6	0.074	0.37, 1.05							0.7	0.209	0.41, 1.22	0.8	0.337	0.43, 1.33
North West	0.6	0.089	0.38, 1.07							0.7	0.164	0.41, 1.16	0.8	0.350	0.43, 1.35
South East	1.8	0.013*	1.12, 2.83							1.3	0.243	0.83, 2.11	1.4	0.266	0.78, 2.41
South South	2.0	0.010*	1.18, 3.29							1.5	0.112	0.91, 2.61	1.5	0.196	0.81, 2.76
South West	1.8	0.039*	1.03, 3.13							1.2	0.512	0.69, 2.12	1.2	0.595	0.62, 2.27
**Community educational level**															
Low (R)															
High	2.3	< 0.001*	1.55, 3.38							1.2	0.433	0.77, 1.84	0.8	0.413	0.50, 1.33
**Community wealth level**															
Low (R)															
High	3.0	< 0.001*	2.11, 4.17							1.8	0.001*	1.30, 2.59	1.2	0.443	0.72, 2.13

R- reference variable; * Statistically significant; CI – confidence interval; p – *p*-value

*Model 0 is the empty model showing crude/unadjusted rates; Model 1 adjusted for individual characteristics alone; Model 2 adjusted for the household characteristics alone; Model 3 adjusted for community characteristics alone; while Model 4 is the full model that adjusted for all the explanatory variables*

## Discussion

To the best of the knowledge of the authors, this study presents de-novo nationally representative data on overweight and obesity among female adolescents in Nigeria. Another strength of the study is how it controlled for a wide range of individual, household and community factors while identifying the determinants of overweight/obesity among the respondents.

The study found that about 10% of the female adolescents were overweight/obese. While some of the existing studies reported similar prevalence rate of obesity among Nigerian adolescents [19, 20, 26–31], a systematic review in Nigeria put the range between 1 − 11.4% [17]. Comparison to the existing studies in Nigeria is challenging because of the varying methodologies, and reference values used to define overweight and obesity, which might be responsible for the very wide range of the reported prevalence rates of adolescent obesity in Nigeria. While the prevalence rate in the present study is lower than what has been found in many high income countries [32, 33], it is similar to what was has been by Studies from other LMIC [6, 34]. The implication of this finding is that the prevalence rate of overweight/obesity among female adolescents in Nigeria is relatively high, despite the high burden of under-nutrition that has also been reported among children and adolescents in Nigeria [35]. 

While the prevalence of overweight/obesity among female adolescents in Nigeria may seem relatively low compared to the global average, i.e10% vs. 18% [5], some factors make this prevalence a cause for concern. Firstly, the prevalence of overweight/obesity among female adolescents (10.2%) is 500% more than that of female under-five children (1.9%), in the same NDHS, 2018 survey [25]. This rapid rise in the prevalence of overweight/obesity among adolescents, and female adolescents particularly needs to be properly investigated and addressed. Furthermore, while overweight/obesity has negative health implications for both sexes, it is particularly more so among females and their unborn child [23, 24]. Female obesity increases the risk of cardiovascular disease, type-2 diabetes, digestive and psychological disorders, maternal morbidity and mortality [12, 14]. It also increases the risk of congenital abnormalities, macrosomia and death in newborn children of obese mothers [36]. 

Another point of concern is the inequalities in the distribution, such that the rate in the South-South region and the richest households is 17% (close to the global average of 18%), while the poorest and poorer households have less than 3%, and rural communities, North-east and North-west regions have a rate of 6%. These inequalities have been similarly reported by previous similar studies [35, 37], and may be a reflection of deeper inequalities existing in the socio-economic and regional structures in Nigeria that need to be addressed. The implication of this is that blanket interventions may not work for the whole country/sub-regions, hence proposed interventions will need to be contextual [18, 38]. 

After controlling for possible individual, household and community level confounding factors, the only factor that retained its statistically significant association with overweight/obesity was the wealth index. The finding of this study corroborates the findings of several previous studies which have found that socio-economic status, measured by the wealth index in this study, is a determinant of overweight/obesity among different age groups [39–41]. However, other studies done within and outside Nigeria have reported other determinants, in addition to household wealth index [18, 41–43], which in this study turned out to be merely confounders. This finding is important for policy makers and stakeholders, because it implies that it may be more effective (including cost-effectiveness) for interventions against overweight and obesity among female adolescents in Nigeria to target adolescents from richer/richest households.

A major limitation of this study is the exclusion of adolescents aged 10–14 years, because this would have given a broader perspective about female adolescent overweight/obesity in Nigeria. Another limitation of this study is reporting and recall bias, particularly the 24-hour dietary recall and other retrospective data relying on memory of a past event. Furthermore, there is an on-going debate on the use of BMI as a measure of obesity such that BMI is described as only a measure of excessive weight rather than excessive body fat. BMI purportedly does not differentiate excess fat, muscle, or bone mass nor provide any indication of the distribution of fat among adolescents. However, BMI was used here because it is still the most commonly used measure [44]. The NDHS contains the most comprehensive Nigerian data on individual, household and community factors [25], and this makes it possible to identify determinants of adolescent overweight/obesity at these levels. However, the NDHS 2018 that was used for this study is a little dated now, even though it is the most current NDHS in Nigeria presently. This is especially so because of the effect of COVID pandemic (which occurred after this study) on the socio-economic factors, which might have affected the present reality as regards overweight/obesity among female adolescents in Nigeria.

## Conclusion

The study found the prevalence of overweight/obesity to be 10.2%, and also found geographical and socio-economic inequalities in the distribution of overweight/obesity among female adolescents in Nigeria. After controlling for possible confounders at individual, household and community levels, the household wealth index remained the only factor with a statistically significant association with overweight/obesity among the respondents. Efforts at addressing overweight and obesity among female adolescents in Nigeria should target those from the richer/richest households.

## Data Availability

The datasets used and/or analyzed during the current study are freely available at the demographic and health survey (DHS) website (https://dhsprogram.com/).

## Abbreviations

BMI: Body mass index
CI: Confidence interval
EA: Enumeration Area
HIC: High Income Countries
LGA: Local Government Areas
LMIC: Low- and middle-income countries
NDHS: Nigeria Demographic and Health Survey
OR: Odds ratio
PSU: Primary Sampling Unit
SD: Standard Deviation
WHO: World Health Organization
